# FIN-EGFR: real-world treatment outcomes by EGFR mutational subtypes in NSCLC in Southern Finland

**DOI:** 10.2340/1651-226X.2026.46295

**Published:** 2026-09-23

**Authors:** Aija Knuuttila, Monica H. Ekblom, Lalli O. Nurmi, Anna Bonin, Eija K. Heikkilä, Maria Silvoniemi

**Affiliations:** a Department of Pulmonary Medicine, Heart and Lung Center, and Cancer Center, Helsinki University Central Hospital, Helsinki, Finland; b Johnson & Johnson, Espoo, Finland; c Nordic Healthcare Group, Espoo, Finland; d Johnson & Johnson, Solna, Sweden; e Department of Respiratory Medicine, Turku University Central Hospital, Turku, Finland

**Keywords:** Non-small-cell lung, receptor, epidermal growth factor, tyrosine kinase inhibitors, overall survival, metastasis, early-stage, advanced-stage

## Abstract

**Background and purpose:**

The aim of the study was to compare overall survival (OS) and time to next treatment (TTNT) of epidermal growth factor receptor (EGFR) mutation positive Non-Small Cell Lung Cancer (NSCLC) patients from start of first therapy, by EGFR mutational sub-type, disease stage at first diagnosis (early vs. advanced stage), first treatment and metastatic sites.

**Patient/material and methods:**

This retrospective observational study utilized patient-level data from hospital data lakes at two university hospital districts in Southern Finland on EGFR-mutation-positive NSCLC patients diagnosed between 2017 and 2023. OS and TTNT were analysed from start of first therapy using Kaplan–Meier, multivariable Cox proportional hazard models and log-rank test.

**Results:**

Among 544 EGFR-mutant NSCLC patients, 42% had early-stage disease. In early-stage NSCLC, OS did not differ significantly between Del19 and L858R mutations, and surgery was the only initial treatment associated with improved OS, with Del19 providing no additional survival benefit. In advanced-stage NSCLC, L858R mutation was a risk factor for shorter OS compared to Del19 (hazard ratio [HR]: 2.01 95% confidence interval [CI]: 1.34–3.02, *p* = 0.001). First-line third-generation tyrosine kinase inhibitor (TKI) showed improved OS compared to first-generation TKI (HR: 0.46 95% CI: 0.28–0.78, *p* = 0.004). Younger patients more often had central nervous system (CNS) and bone metastases. CNS and liver metastases at diagnosis were linked to shorter OS (5.0 and 8.1 months, respectively).

**Interpretation:**

EGFR subtype did not affect outcomes in early-stage NSCLC, while Del19 mutations and first-line third-generation TKIs were associated with better outcomes in advanced disease. Younger patients had higher rates of CNS and bone metastases, which were associated with shorter OS.

## Introduction

The hospital data lakes in Finland enable unique opportunities for investigating ‘real-world’ outcomes in patients with epidermal growth factor receptor (EGFR) mutated non–small-cell lung cancer (NSCLC) by combining hospital electronic health records (EHRs) with national registers. Standardized national EGFR tyrosine kinase inhibitor (TKI) reimbursement, centralized healthcare, and high EGFR testing rates further strengthen the Finnish setting for EGFR-mutated NSCLC treatment outcome research.

For early-stage EGFR-mutated NSCLC, surgery is the best option. However, 30–55% of patients experience recurrence and ultimately die of their disease despite radical resection [1–3]. Perioperative treatment, including neoadjuvant and adjuvant therapies, can offer additional benefits. Historically, this has primarily involved chemotherapy, which, however, has resulted in only modest improvements in OS: 5.4% with adjuvant and 5% with neoadjuvant therapy [4, 5].

For patients with advanced NSCLC and sensitizing EGFR mutations including exon 19 deletions (Del19) or L858R substitution (L858R), current guidelines recommend first-line EGFR TKI combination or monotherapy, irrespective of clinical parameters [6]. In Finland, the first-generation TKIs erlotinib and gefitinib received reimbursement in November 2007 and November 2010, respectively, the second-generation afatinib in December 2016, and the third-generation TKI osimertinib received reimbursement in first-line in October 2020. During the study period only TKI-monotherapy was available for advanced disease. For patients with EGFR Exon 20 insertion mutations the current recommendations state that amivantamab (EGFR-MET bispecific antibody) ± chemotherapy should be used [7] while only chemotherapy was available during the study period. Additionally, osimertinib received reimbursement for adjuvant therapy after tumor resection in May 2022 concerning Del19 and L858R mutation positive NSCLC.

Despite the development of targeted therapies for EGFR mutated NSCLC, long-term prognosis remains limited due to acquired resistance [8]. Compared to first- and second-generation TKIs, first-line osimertinib has shown longer overall survival (OS) in clinical trials (38.6 vs. 31.8 months). However, the subgroup analysis showed that this benefit was seen only in patients with Del19 mutations, as those with L858R showed no OS difference [9].

Metastatic spread to distant organs accounts for most cancer-related deaths. In EGFR mutation–positive NSCLC, the incidence of central nervous system (CNS) metastases is higher than in EGFR wild-type disease. Approximately 30–50% of the EGFR –mutation-positive patients develop CNS metastases within 5 years of diagnosis [10]. The third-generation EGFR TKI has shown improved CNS penetrance over earlier-generation TKIs [11, 12]. Liver and bone metastases are also common in lung cancer and are linked to shorter survival [13]. However, ‘real-world’ studies on these patients, their characteristics, and treatment outcomes remain scarce.

We have previously shown in the FIN-EGFRprint study that OS and time to next treatment (TTNT) improved over time (2010–2023), coinciding with the transition from first- to third-generation TKIs among patients with common EGFR mutations (Del19 and L858R) [14]. This study, utilizing the same Finnish dataset, extends these findings by investigating OS and TTNT according to EGFR mutation subtype, including not only the common Del19 and L858R mutations but also less-studied uncommon EGFR mutations. In addition, we evaluate treatment outcomes in both early- and advanced-stage disease, which, to our knowledge, have not previously been investigated within the same dataset. Furthermore, we evaluate the impact of CNS and liver metastases on treatment outcomes.

## Patients/material and methods

### Study design

This retrospective observational study included all NSCLC patients with defined and confirmed EGFR mutations from Finland’s two largest university hospitals – Helsinki University Hospital (HUS) and Turku University Hospital (TYKS). These patients represent ~41% of all NSCLC cases in Finland. Patients were followed from baseline (1-year prior diagnosis) until death or the end of the study period.

### Data sources

Patients’ medical records were collected from EHRs in the hospital data lakes of HUS and TYKS. EGFR TKI purchases for patients who received reimbursement for EGFR TKIs (gefitinib, erlotinib, afatinib, or osimertinib) were collected from the Special Reimbursement Registry of the Social Insurance Institution of Finland. Hospital data lakes are data repositories that integrate patient-level information from multiple hospital information systems and include both structured and unstructured clinical data from healthcare visits and hospitalizations. So, this data is not collected the traditional way from manually kept and updated patient registries. Prior to analysis pseudonymization was performed by Findata – the data permit authority for the social and healthcare sector under the Act on the Secondary Use of Health and Social Data (552/2019).

### Study population

This study included adult patients (≥18 years of age) who were diagnosed with NSCLC (excluding international classification of diseases [ICD]-10 codes C34.X6 and C34.X7) during 01 January 2017–30 September 2023 and had defined and confirmed EGFR mutations. Patients were followed until the end of the study period, 31 December 2023. A minimum follow-up period of 3 months from the NSCLC diagnosis was required for inclusion.

### EGFR mutational subtypes

EGFR mutation data were collected from EHRs, and the EGFR mutational subtypes were classified according to groups used in prior studies [15–17]. The EGFR mutational groups were stratified into (1) exon 19 deletions (Del19), (2) exon 21 L858R substitutions (L858R); (3) exon 20 insertion (Exon20ins); (4) Major uncommon mutations – G719X, L861Q, or S768I; (5) other rare EGFR mutations, grouped under ‘Other uncommon’; and (6) patients with more than one EGFR mutation, categorized as ‘Multiple’. If the T790M mutation was recorded simultaneously at diagnosis along with any other EGFR mutation, the patient was included in the Multiple-group. The proportion of EGFR-untested patients and the incidence of EGFR mutations were estimated among patients with NSCLC, excluding patients with small cell lung cancer (C34.X6) from these analyses.

### Patients diagnosed at early and advanced disease stage

The patients were stratified according to disease stage at first NSCLC diagnosis (early or advanced stage) based on the stage records (early stage I–IIIa; advanced stage: IIIb–IV) or first treatment received after NSCLC diagnosis in case stage records were not available at diagnosis in EHRs. Stages I–IIIa were categorized as early-stage disease, as these stages have no distant metastases and may still be approached with curative intent. Stage IIIb represents locally advanced disease that is generally beyond curative-intent therapy and was therefore categorized as advanced-stage disease together with stage IV.

### NSCLC-specific treatments

Early-stage treatments included surgery; radiotherapy with curative intent; surgery or radiotherapy with neoadjuvant or adjuvant chemotherapy (Surgery/radiotherapy + adjuvant/neoadjuvant CT); or Surgery followed by adjuvant third generation TKI (osimertinib) (Surgery + adjuvant 3rd gen TKI). Advanced-stage treatments included any TKI, chemotherapy, palliative radiotherapy or other treatments including immune therapy administered as first-line therapy. TKI treatments included first generation TKIs erlotinib and gefitinib, second generation TKI afatinib and third generation TKI osimertinib. First- and second-generation TKIs were available throughout the study, as these reimbursements were received before 2017; reimbursement for third-generation TKI was received in October 2020. The patients receiving chemotherapy ± 2 months from surgery or radiotherapy were grouped into the early-stage population while those receiving chemotherapy as monotherapy were grouped into the advanced-stage population.

### Metastasis

CNS, liver, and bone metastasis records were extracted from physicians’ notes in the EHR using text mining and subsequently validated manually. A keyword list was developed to capture terms indicating cancer diagnosis, disease stage, or metastatic spread, and was deliberately specified with high sensitivity at the expense of specificity, so as to retain all potential positive cases while accepting a substantial number of false positives. All records flagged by the keyword search were subsequently reviewed manually by hand validation, and were classified as positive only where the clinician had explicitly documented a diagnosis of metastatic cancer; ambiguous or non-confirmatory mentions were excluded. Metastases were classified as Early if records of the specific metastasis occurred within 3 months before or after the NSCLC diagnosis, indicating the metastasis was present at baseline. If records were present more than 3 months after diagnosis, the metastases were classified as Late, indicating that the metastases developed after baseline. The reference groups comprised of advanced-stage patients without the respective metastasis (non-groups). Accordingly, the following groups were defined: Early-/Late-/Non-CNS, Early-/Late-/Non-Liver, and Early-/Late-/Non-Bone.

### Outcome and statistical analysis

To ensure patient anonymity, results are only reported for data from groups of at least five patients. Descriptive statistics are presented as the mean (standard deviation) for continuous variables and as a number (%) for categorical variables. The patients who received no active treatment, palliative radiotherapy only, or a curative-intent modality despite advanced stage were excluded from the OS and TTNT analyses. OS and TTNT were analysed using Kaplan–Meier (KM) estimates, starting from the first treatment following the NSCLC diagnosis. Differences between groups in the KM analysis were examined using the log-rank test. Multivariable Cox models were adjusted for sex, age group, EGFR subtype and first treatment, plus metastatic site at diagnosis in advanced-stage models. Covariates were prespecified in the study protocol; no directed acyclic graph or data-driven selection was used, and stage was handled by fitting separate models per stage. Eastern Cooperative Oncology Group (ECOG) and smoking were unavailable for most patients and thus not included in the model. No covariate values were missing and no imputation was performed. Proportional hazards held in every model (scaled Schoenfeld residuals, global cox.zph *p* > 0.05). Hazard ratios are reported as adjusted associations. Analyses were performed using *R* statistical software.

### Ethical considerations

The study was conducted in compliance with the Declaration of Helsinki. The study was approved by the national data permit authority, Findata (THL/2280/14.02.00/2023). According to Finnish legislation, separate ethical approval is not required for retrospective registry studies conducted under the Secondary Use of Health Data Act.

## Results

### Patients’ characteristics

**Figure 1:**
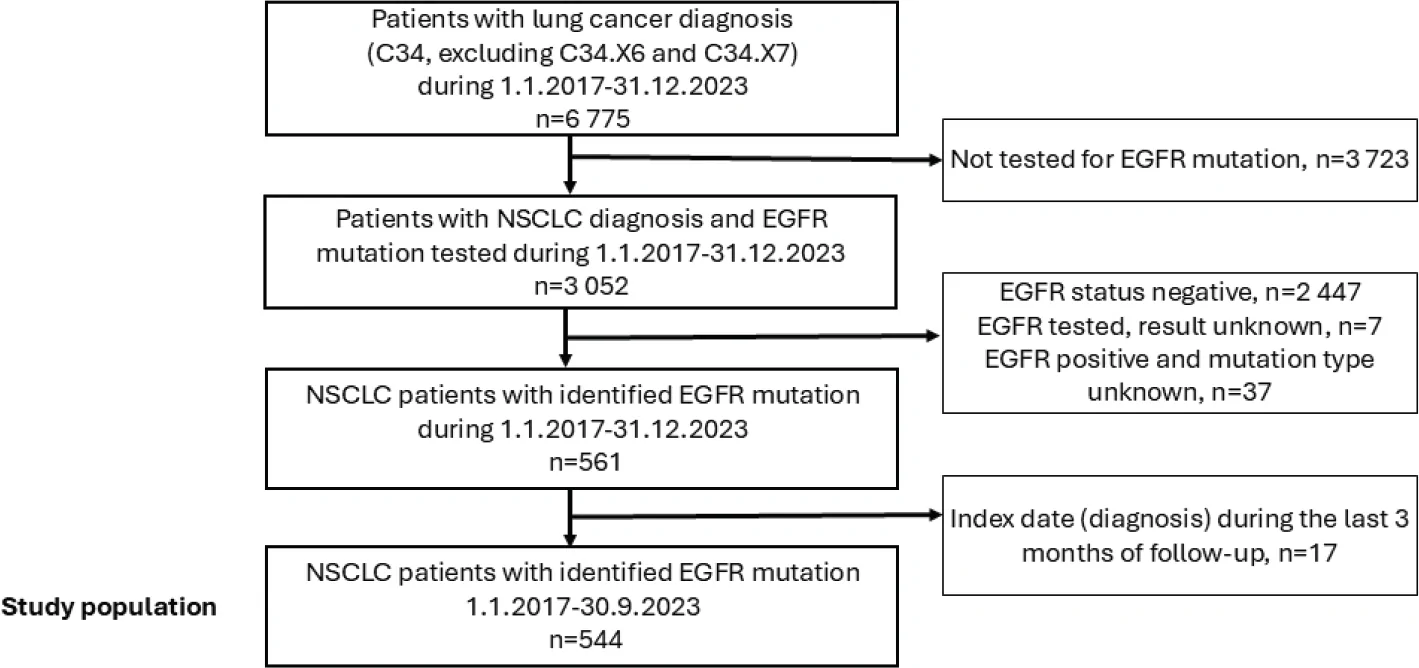
Flow chart for the study population. NSCLC: Non-Small Cell Lung Cancer; EGFR: epidermal growth factor receptor.

A total of 544 NSCLC patients were found to have an identifiable EGFR mutation and were included in the cohort. These included both patients diagnosed at early-stage and advanced-stage (Figure 1). An EGFR test result was identified for 3052 of 6775 incident NSCLC patients (45%).

The patients without EGFR testing (*n* = 3723), the histological subtypes were squamous cell carcinoma (28%, C34.X1), adenocarcinoma (16%, C34.X2–X3), unspecified histology (11% C34.X9), other NSCLC (3%, C34.X5), multiple histological findings (1%) and no histological confirmation (41%, C34.X0). The cohort represented 8% of incident NSCLC patients, annual share stable at 7–9% during the study period.

Baseline characteristics of the whole cohort including patients of all stages are summarized in Table 1. The cohort had a median age of 71.6 years and 68% were women, 87% had adenocarcinoma and 2% squamous cell carcinoma. Forty-two per cent of the patients were diagnosed in early-stage and 55% in advanced-stage. Three per cent of the patients had no recorded stage information and did not receive any active lung cancer treatment; therefore, they could not be stratified into early or advanced-stage groups. Median age at diagnosis was similar for both early and advanced-stage patients.

**Table 1:** Patients’ characteristics.

Characteristics	Del19	L858R	Exon20ins	Multiple	Major uncommon	Other uncommon	All
**Share by mutation, *n* (%)**	220 (40%)	180 (33%)	30 (6%)	37 (7%)	28 (5%)	49 (9%)	544 (100%)
**Female, *n* (%)**	151 (69%)	129 (72%)	20 (67%)	30 (81%)	18 (64%)	23 (47%)	371 (68%)
**Age, median (IQR)**	71.2 (62.3–77.1)	70.9 (65.6–76.7)	72.2 (67.0–76.1)	69.0 (63.8–77.6)	74.1 (71.1–82.2)	72.1 (66.6–77.7)	71.6 (64.8–77.5)
**Age groups, years, *n* (%)**							
< 65	67 (30%)	40 (22%)	6 (20%)	12 (32%)	6 (21%)	9 (18%)	140 (26%)
65–74	79 (36%)	79 (44%)	16 (53%)	13 (35%)	9 (32%)	22 (45%)	218 (40%)
75–79	33 (15%)	32 (18%)	4 (13%)	7 (19%)	# (~10%)	12 (25%)	91 (17%)
≥ 80	41 (19%)	29 (16%)	4 (13%)	5 (14%)	# (~36%)	6 (12%)	95 (17%)
**Histology, *n* (%)**							
Adenocarcinoma	194 (88%)	164 (91%)	25 (83%)	# (~90%)	# (~90%)	30 (61%)	472 (87%)
Other	26 (12%)	16 (9%)	5 (17%)	# (~10%)	# (~10%)	19 (39%)	72 (13%)
**Stage, *n* (%)**							
Advanced stage (IIIb–IV)	120 (55%)	101 (56%)	19 (63%)	19 (51%)	13 (46%)	27 (55%)	299 (55%)
Early stage (I–IIIa)	# (~45%)	# (~44%)	# (~34%)	# (~42%)	15 (54%)	# (~38%)	231 (42%)
Missing	< 5	< 5	< 5	< 5	0	< 5	14 (3%)
**Comorbidities, *n* (%)**							
Other cancer	40 (18%)	32 (18%)	5 (17%)	7 (19%)	12 (43%)	9 (18%)	105 (19%)
**Metastatic location in advanced stage at diagnosis *n* (%)**	120 (100%)	101 (100%)	19 (100%)	19 (100%)	13 (100%)	27 (100%)	299 (100%)
CNS	28 (23%)	23 (23%)	< 5	< 5	< 5	11 (41%)	70 (23%)
Liver	27 (23%)	18 (18%)	< 5	8 (42%)	< 5	6 (22%)	62 (21%)
Bone	50 (42%)	45 (45%)	< 5	5 (26%)	< 5	8 (30%)	115 (38%)
CNS, liver or bone or in combination	70 (32%)	56 (31%)	7 (23%)	12 (63%)	5 (39%)	16 (59%)	166 (56%)

If the patient number is less than five (< 5), it is displayed as ‘< 5’. If the patient number is hidden (#), it means that displaying it would allow the calculation of the exact patient number. This is done to preserve participant anonymity and comply with the General Data Protection Regulation (GDPR). CNS: central nervous system; EGFR: epidermal growth factor receptor; IQR: interquartile range; Del19: exon 19 deletions; L858R: exon 21 L858R substitutions; Exon20ins: exon 20 insertion; Multiple: more than one EGFR mutation; Major uncommon mutations: G719X, L861Q, or S768I; Other uncommon: rare EGFR mutations.

The smoking status was available for 43% of the patients. Of those, 11% were current smokers, 22% were ex-smokers and 67% had never smoked. The most common EGFR mutations were Del19 (40%) and L858R (33%). Exon20ins mutations were found in 6% of the patients. Nineteen per cent of the patients had a history of a previous other cancer diagnosis (mainly breast or prostate cancer). Of the advanced-stage patients (*n* = 299) 23% had CNS metastasis, 21% had liver metastasis and 38% had bone metastasis at diagnosis.

### Treatment progression from first therapy for patients diagnosed at early or advanced stage

#### Early-stage patients

**Figure 2:**
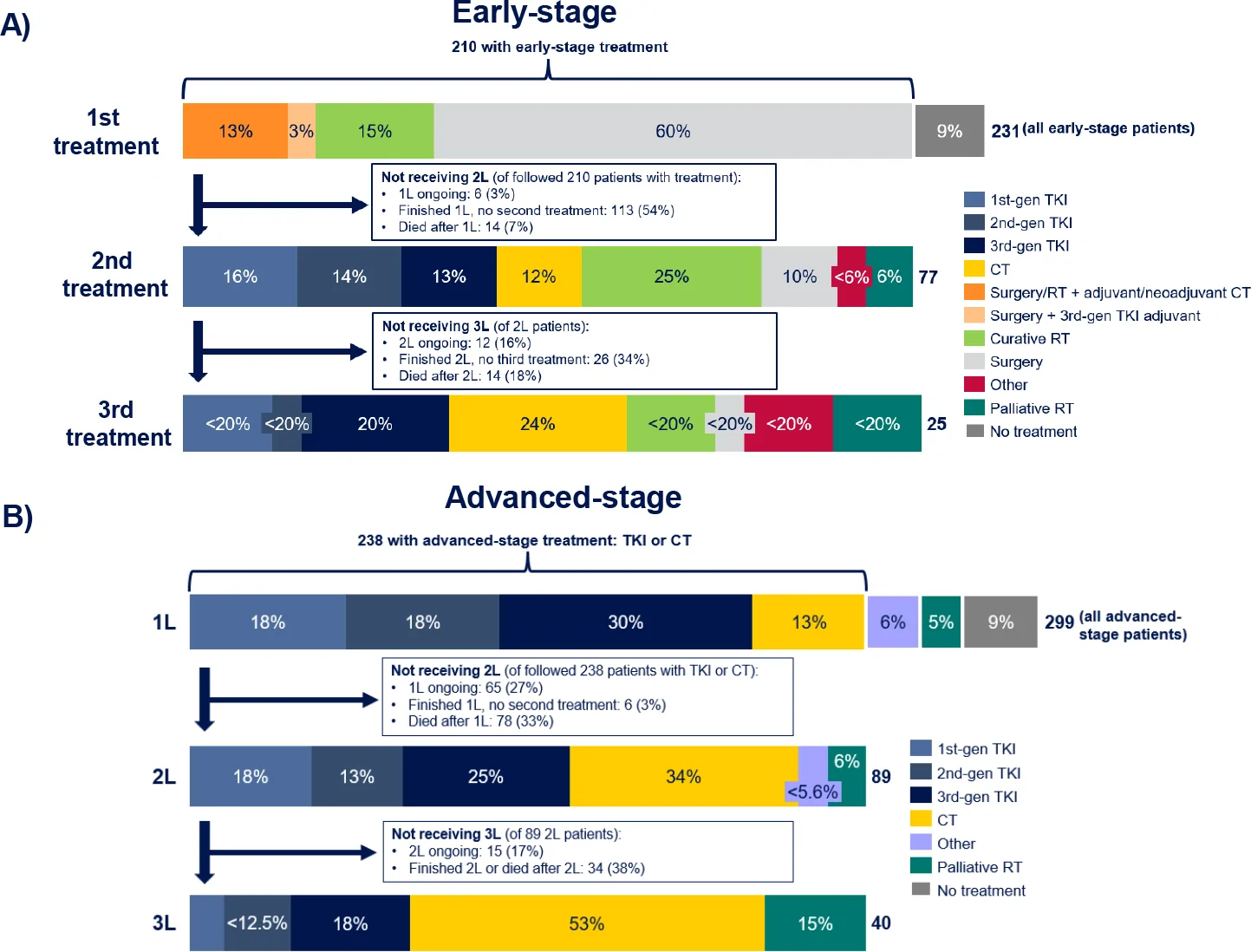
Treatment progression for patients diagnosed at (A) early-stage and (B) advanced-stage for EGFR mutated NSCLC. TKI: tyrosine kinase inhibitor; CT: chemotherapy; RT: radiotherapy; NSCLC: Non-Small Cell Lung Cancer; EGFR: epidermal growth factor receptor.

In early-stage patients, surgery was the most common first treatment (60%), followed by curative radiotherapy (15%). Thirteen per cent were treated with surgery or radiotherapy combined with adjuvant or neoadjuvant chemotherapy. A small proportion were treated with adjuvant third generation TKI following surgery and 9% received no active treatment. During follow-up, 37% received additional therapy, most commonly TKI (43%), while 54% received no further treatment (Figure 2A).

#### Advanced-stage patients

In advanced-stage patients, third-generation TKI was the most common first-line treatment (30%), followed by first- and second-generation TKIs (18% each). Most third-generation TKI users (94%) had common EGFR mutations (Del19: 45%, L858R: 49%). Chemotherapy was used in 13%, while 10% received no active lung cancer treatment. Of the untreated patients, 52% had a Del19 or L858R mutation, 22% had an Exon20ins mutation, and 26% had a major or other uncommon mutation. Among treated patients, 37% received second-line therapy, most commonly TKIs (56%), especially third-generation TKI (26%) and one-third of the patients received chemotherapy as their second-line treatment. One-third died without receiving second-line treatment, while 27% remained on first-line therapy at follow-up end (Figure 2B).

### OS by EGFR mutational subtype and first treatment

OS was analyzed by EGFR mutational subtype and first treatment type. The patients who received no active lung cancer treatment (*n* = 48), palliative radiotherapy only (*n* = 15), or a curative-intent modality despite advanced stage (*n* = 19) were excluded from the OS and TTNT analyses, leaving 210 early-stage and 238 advanced-stage patients for the OS analysis.

Median OS for early-stage patients was not reached whereas the median OS for patients diagnosed in advanced-stage was 23.0 months (95% CI: 18.0–29.0). The 5-year survival probability was 70.3 and 15.8%, respectively.

#### Early-stage patients

In early-stage patients, median OS was not reached in most EGFR subgroups or first therapy groups, except for radiotherapy with curative intention (57.6 months) (Supplementary Figure 1). During the follow up 35 deaths occurred among 210 patients. In multivariable analysis, only first treatment type was associated with OS, with surgery linked to longer OS compared with radiotherapy with curative intent or radiotherapy/surgery combined with neo- or adjuvant chemotherapy (Supplementary Table 1). Among patients who received surgery as first therapy, OS was similar between Del19 and L858R mutations. From these patients 16 died from 138 patients during follow up (Supplementary Table 2).

#### Advanced-stage patients

**Figure 3:**
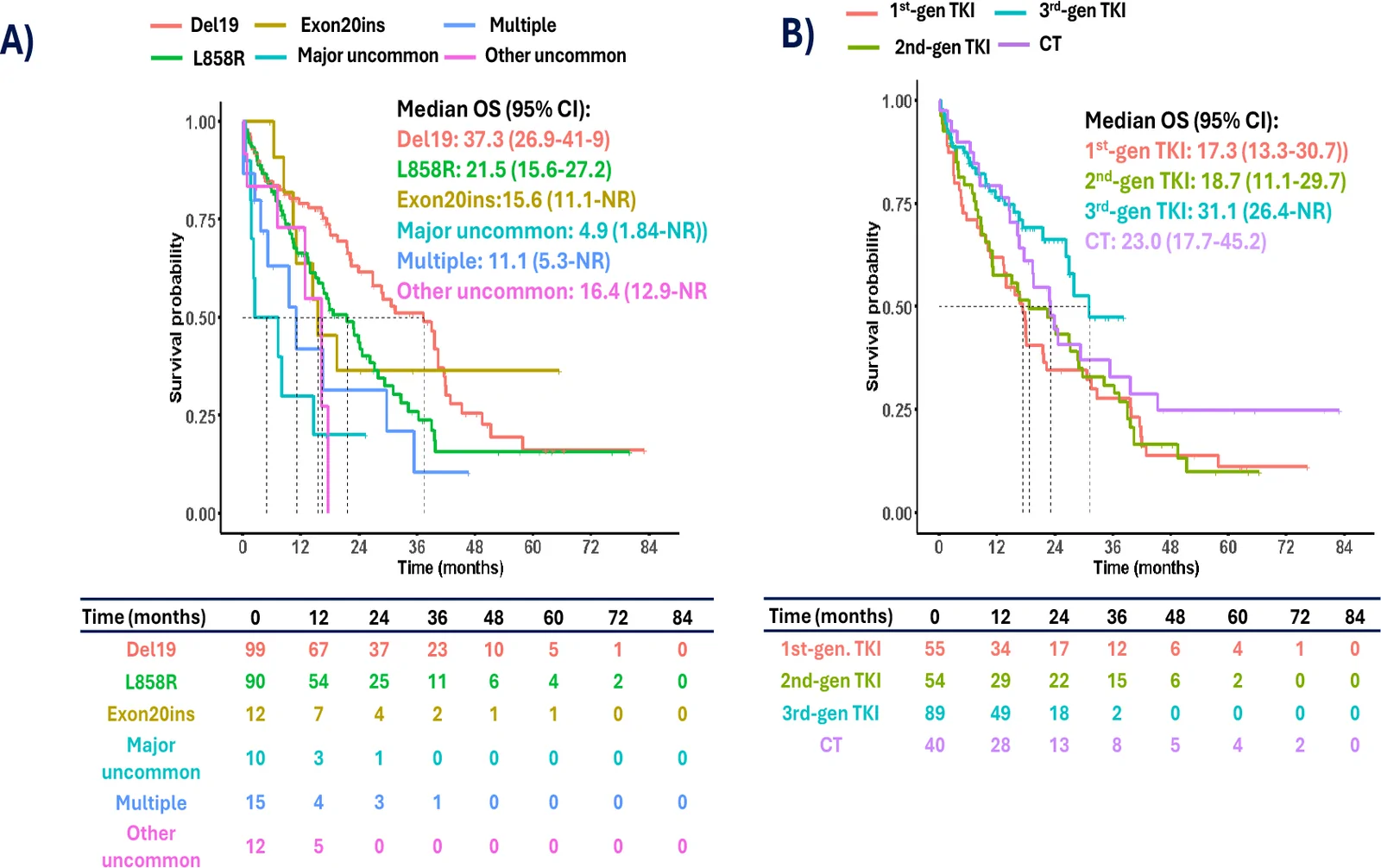
Overall survival (OS) for patients diagnosed at advanced-stage EGFR mutated NSCLC by (A) EGFR mutational group or (B) treatment type. Del19: exon 19 deletions; L858R: exon 21 L858R substitutions; Exon20ins: exon 20 insertion; Multiple: more than one EGFR mutation; Major uncommon mutations: G719X, L861Q, or S768I; Other uncommon: rare EGFR mutations; TKI: tyrosine kinase inhibitor; CT: chemotherapy; NSCLC: Non-Small Cell Lung Cancer; EGFR: epidermal growth factor receptor.

In advanced-stage patients, median OS was 37.3 months for Del19 mutations and 21.5 months for L858R mutations. First-line third-generation TKI was associated with the longest OS (31.1 months), while first-generation TKIs had the shortest (17.3 months) (Figure 3). During the follow up 141 deaths occurred among 238 patients. In multivariable analysis, Del19 mutations, and female sex were linked to longer OS, whereas CNS and liver metastases were associated with shorter OS. For treatment types both third-generation TKI and chemotherapy were linked to longer OS compared to first-generation TKI (Table 2).

**Table 2:** Multivariable Cox proportional hazards regression results for OS among patients diagnosed at an advanced stage (n = 238).

Variable	Category	*n* (%)	HR	95% CI	*p*
Sex	Male	78 (32.8)	-	-	-
	Female	160 (67.2)	0.62	0.43–0.89	*p* = 0.009
Age group	< 65	71 (29.8)	-	-	-
	65–75	94 (39.5)	0.97	0.62–1.50	*p* = 0.876
	> 75	73 (30.7)	1.16	0.70–1.92	*p* = 0.569
EGFR mutation	Del19	99 (41.6)	-	-	-
	L858R	90 (37.8)	2.01	1.34–3.02	*p* = 0.001
	Exon20ins	12 (5.0)	1.93	0.83–4.50	*p* = 0.126
	Major uncommon	10 (4.2)	6.30	2.82–14.10	*p* < 0.001
	Multiple	15 (6.3)	3.39	1.57–7.28	*p* = 0.002
	Other uncommon	12 (5.0)	3.47	1.30–9.30	*p* = 0.013
First-line treatment	1st-gen TKI	55 (23.1)	-	-	-
	2nd-gen TKI	54 (22.7)	0.73	0.45–1.19	*p* = 0.210
	3rd-gen TKI	89 (37.4)	0.46	0.28–0.78	*p* = 0.004
	Chemotherapy	40 (16.8)	0.45	0.25–0.84	*p* = 0.011
Metastasis location	Not CNS or liver	163 (68.5)	-	-	-
	CNS	34 (14.3)	3.32	1.26–8.75	*p* = 0.015
	Liver	33 (13.9)	2.49	1.51–4.12	*p* < 0.001
	CNS and liver	8 (3.4)	2.10	1.27–3.49	*p* = 0.004

The proportional hazards assumption was assessed and showed no evidence of violation (global *p* = 0.096). Del19: exon 19 deletions; L858R: exon 21 L858R substitutions; Exon20ins: exon 20 insertion; Multiple: more than one EGFR mutation; Major uncommon mutations: G719X, L861Q, or S768I; Other uncommon: rare EGFR mutations; TKI: tyrosine kinase inhibitor; CNS: central nervous system; EGFR: epidermal growth factor receptor; OS: overall survival.

### TTNT by EGFR mutational subtype and first treatment

#### Early-stage patients

In early-stage patients, median TTNT was not reached for EGFR mutational subtype or first treatment (Supplementary Figure 1). During the follow up 91 patients initiated new treatment among 210 patients. Female sex and surgery as first treatment were associated with longer TTNT, while Del19 mutations showed no TTNT advantage over L858R mutations (Supplementary Table 3).

#### Advanced-stage patients

**Figure 4:**
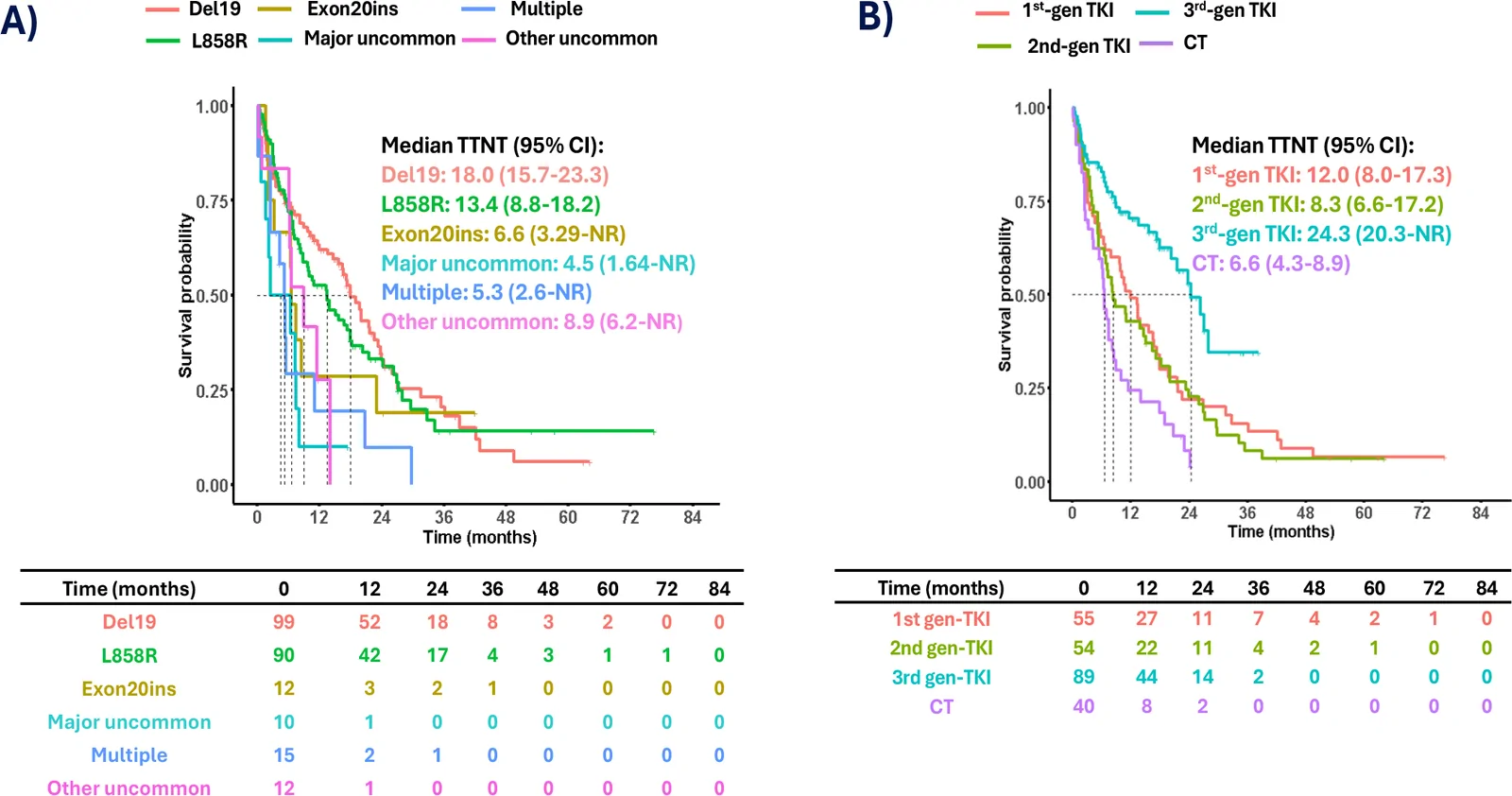
Time to next treatment (TTNT) for patients diagnosed at advanced-stage EGFR mutated NSCLC by (A) EGFR mutational group or (B) treatment type. Del19: exon 19 deletions; L858R: exon 21 L858R substitutions; Exon20ins: exon 20 insertion; Multiple: more than one EGFR mutation; Major uncommon mutations: G719X, L861Q, or S768I; Other uncommon: rare EGFR mutations; TKI: tyrosine kinase inhibitor; CT: chemotherapy; NSCLC: Non-Small Cell Lung Cancer; EGFR: epidermal growth factor receptor.

In advanced-stage patients, third-generation TKI had longest median TTNT (24.3 months) and chemotherapy shortest (6.6 months) (Figure 4). During the follow up 168 patients initiated new treatment among 238 patients. Third generation TKI was associated with longer TTNT, while CNS and liver metastases predicted shorter TTNT. Del19 mutations were not associated with significantly longer TTNT than L858R mutations (Table 3).

**Table 3:** Multivariable Cox proportional hazards regression results for TTNT among patients diagnosed at an advanced stage (n = 238).

Variable	Category	*n* (%)	HR	95% CI	*p*
Sex	Male	78 (32.8)	-	-	-
	Female	160 (67.2)	0.61	0.43–0.85	*p* = 0.004
Age group	< 65	71 (29.8)	-	-	-
	65–75	94 (39.5)	1.03	0.69–1.54	*p* = 0.889
	> 75	73 (30.7)	1.18	0.75–1.86	*p* = 0.476
EGFR mutation	Del19	99 (41.6)	-	-	-
	L858R	90 (37.8)	1.28	0.89–1.86	*p* = 0.183
	Exon20ins	12 (5.0)	0.96	0.43–2.13	*p* = 0.924
	Major uncommon	10 (4.2)	4.01	1.88–8.56	*p* < 0.001
	Multiple	15 (6.3)	1.95	0.97–3.91	*p* = 0.062
	Other uncommon	12 (5.0)	0.80	0.33–1.93	*p* = 0.619
First-line treatment	1st-gen TKI	55 (23.1)	-	-	-
	2nd-gen TKI	54 (22.7)	0.85	0.54–1.35	*p* = 0.490
	3rd-gen TKI	89 (37.4)	0.45	0.28–0.72	*p* = 0.001
	Chemotherapy	40 (16.8)	1.79	1.02–3.14	*p* = 0.042
Metastasis location at diagnosis	Not CNS or Liver	163 (68.5)	-	-	-
	CNS	34 (14.3)	2.34	1.45–3.79	*p* = 0.001
	Liver	33 (13.9)	1.63	1.02–2.61	*p* = 0.040
	CNS and liver	8 (3.4)	1.70	0.66–4.41	*p* = 0.275

The proportional hazards assumption was assessed showed no evidence of violation (global *p* = 0.131). Del19: exon 19 deletions; L858R: exon 21 L858R substitutions; Exon20ins: exon 20 insertion; Multiple: more than oneEGFR mutation; Major uncommon mutations: G719X, L861Q, or S768I; Other uncommon: rare EGFR mutations; TKI: tyrosine kinase inhibitor; CNS: central nervous system; TTNT: Time to next treatment; EGFR: epidermal growth factor receptor.

### Metastasis

#### Proportion of patients with CNS, liver, and bone metastases

Among 299 advanced-stage patients, 38% had CNS, 31% liver, and 56% bone metastases. At diagnosis, 23% had CNS, 21% liver, and 38% bone metastases, while additional metastases developed more than 3 months later in CNS (11%), liver (8%), and bone (12%) of patients. Among early-stage patients, 5% later developed CNS, 3% liver, and 7% bone metastases.

#### Patient characteristics and first therapy patterns in patients with CNS, liver, and bone metastases

Early-CNS patients were younger than non-CNS patients (69.2 vs. 73.2 years, *p* = 0.0002), with fewer patients aged > 75 years (13 vs. 17%) and > 80 years (6 vs. 25%). No significant age difference was seen between early-liver and non-liver groups (69.3 vs. 71.3 years). Early-bone patients were also younger than non-bone patients (69.5 vs. 72.8 years, *p* = 0.0007), with fewer patients aged > 80 years (14 vs. 23%).

First-line EGFR TKI use was highest in early-bone patients (70%), followed by early-liver (57%) and early-CNS (56%). No active lung cancer treatment was most common in early-liver (16%), compared with early-CNS (9%) and early-bone (6%). Palliative radiotherapy without systemic therapy was used in 25% of early-CNS patients versus 10% of early-liver and early-bone patients.

#### OS in patients with CNS, liver and bone metastasis

**Figure 5:**
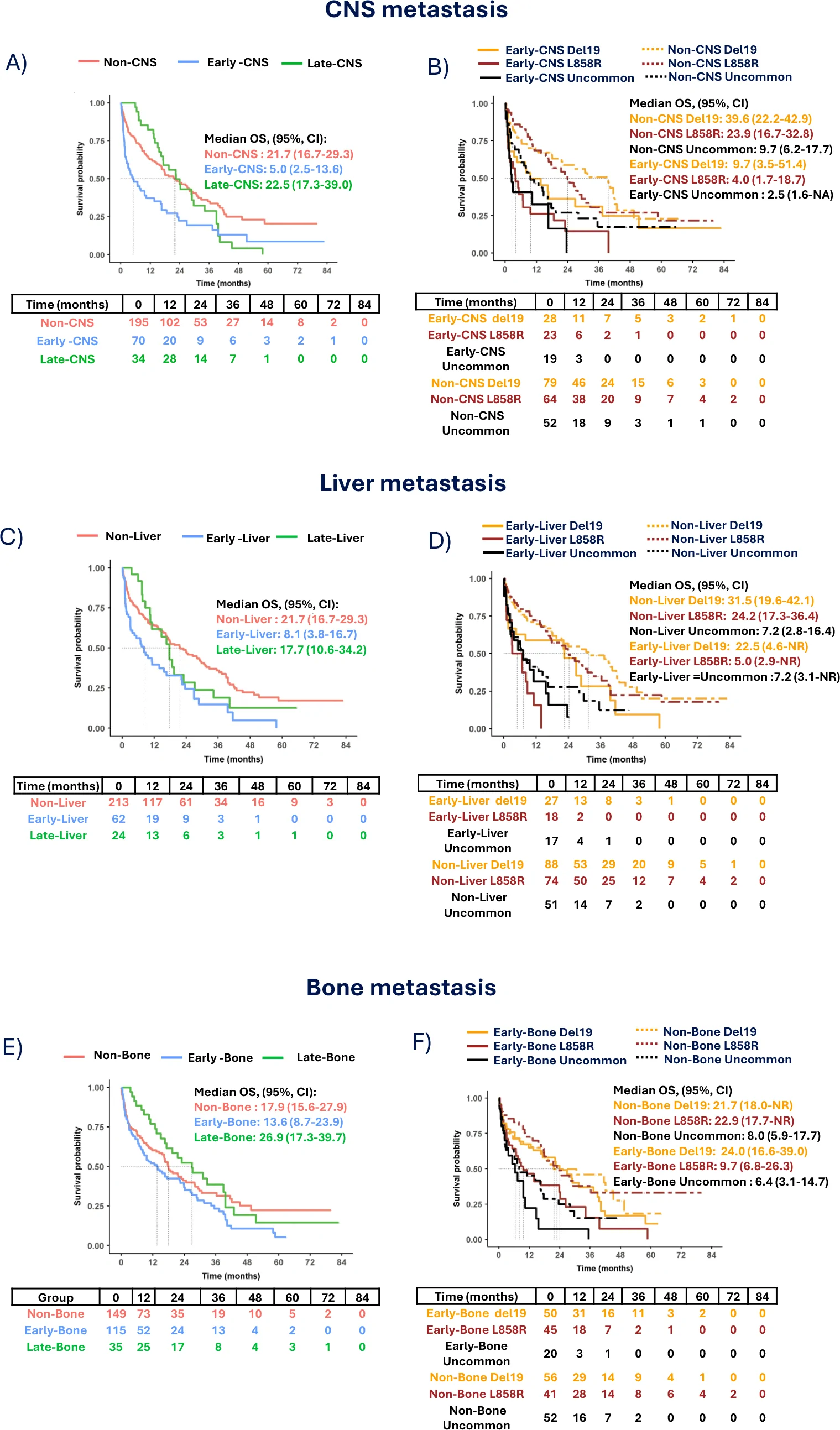
Overal survival (OS) for EGFR-NSCLC patients by CNS, liver or bone metastasis status and by EGFR mutational groups. Del19: exon 19 deletions; L858R: exon 21 L858R substitutions; Uncommon: includes Multiple, Major uncommon, and Other uncommon mutations; CNS: central nervous system; non-group: advanced-stage patients without the respective metastasis; Early-group: respective metastasis occurred within 3 months before or after the NSCLC diagnosis; Late-group: respective metastasis occurred later than 3 months after the NSCLC diagnosis; NSCLC: Non-Small Cell Lung Cancer; EGFR: epidermal growth factor receptor.

Median OS was significantly shorter in Early-CNS and early-liver patients than in patients without these metastases (non-groups), while no difference was seen between early-bone and non-bone groups. Median OS was shortest in early-CNS patients (5.0 months), followed by early-liver (8.1 months) and early-bone (13.6 months) (Figures 5A, 5C, and 5E).

To analyse the effect of EGFR mutational subtype on OS, the Del19 and L858R subtypes were included separately, and Exon20ins, Multiple, Major uncommon, and Other uncommon mutations were combined as ‘Uncommon’. In Early-CNS patients, OS did not differ between EGFR subtypes. In Early-Liver patients, Del19 mutations were associated with longer OS than L858R mutations (22.5 vs. 5.0 months, p=0.027). In early-bone patients, Del19 mutations showed longer OS than uncommon mutations (24.0 vs. 6.4 months, p=0.0018) (Figure 5F).

## Discussion and conclusion

In this retrospective ‘real-world’ study of EGFR-mutated NSCLC, we assessed OS and TTNT by EGFR subtype, first therapy, and metastatic site in early- and advanced-stage disease. In early-stage NSCLC, surgery was associated with longer OS and TTNT, while EGFR subtype had no impact. In advanced-stage disease, third-generation TKIs provided the longest OS and TTNT, and Del19 mutations were associated with better OS than L858R mutations.

In early-stage disease, Del19 mutations did not improve OS compared with L858R mutations, including in surgically treated patients, suggesting EGFR subtype may not influence treatment selection. In the early-stage first-treatment analysis, surgery as the first treatment was associated with longer OS compared with other first treatments. However, further adjustment for factors such as treatment intent, operability, or performance status was not possible due to limited patient numbers within treatment groups and limited availability of this information in the dataset.

For advanced-stage patients, Ramalingam *et al.* found in a Phase 3 trial subgroup analysis, that patients with Del19 mutations had longer OS than those with L858R mutations when treated first-line with osimertinib [9]. Also a ‘real-world’ nationwide study from the Netherlands (*n* = 1019) found that patients with Del19 mutations showed improved OS compared to L858R mutation with first-line TKI treatment [18]. Consistent with these findings, our study also showed significantly longer OS for Del19 compared with L858R and other EGFR mutation subgroups, although numbers in the latter groups were small. The superior outcomes observed with Del19 are most likely due to its higher sensitivity to TKIs compared to other EGFR mutations, as demonstrated also in previous studies [9, 18]. These findings highlight that patients with L858R, Exon20ins, or other EGFR mutations have a particularly high unmet need for novel therapies beyond third-generation TKIs, although it must be noted that treatment outcomes remain limited across the entire advanced NSCLC population.

In a phase 3 trial, first-line osimertinib improved OS versus erlotinib/gefitinib in patients with common EGFR mutations (38.6 vs 31.8 months) [9]. Similarly, in our study, third-generation TKI was associated with significantly longer OS than first-generation TKIs (median OS, 31.1 vs. 17.3 months, respectively, by unadjusted KM analysis), and this association remained significant in the multivariable Cox proportional hazards regression analysis (adjusted HR, 0.46). However, not all real-world studies found OS advantage for third-generation TKIs. A nationwide registry study from the Netherlands showed no difference in OS results between TKIs in patients with common EGFR mutations (gefitinib:19.7, erlotinib: 23.2, afatinib:23.3 vs. osimertinib: 22.8 months). The subgroup analysis, however, showed that the OS benefit with osimertinib was restricted to patients with Del19 mutations and baseline CNS metastasis [18]. Likewise, a UK study (*n* = 336) found no OS difference between third- and earlier-generation TKIs (16.6 vs. 16.9 months), but included 20% uncommon mutations and did not analyze Del19 and L858R separately [19]. Altogether, these findings suggest that OS differences between TKI generations may depend on the proportion of Del19-mutated patients, who generally have better outcomes with third-generation TKIs.

TTNT results were consistent with OS findings, with third-generation TKIs showing longer TTNT than first-generation TKIs (24.3 vs. 12.0 months), highlighting the importance of first-line treatment choice. A recent European real-world study (*n* = 1646) reported TTNTs of 18.8 months for third-generation TKIs and 6.6 months for chemotherapy in advanced EGFR-mutated NSCLC [20]. In earlier years, it was more common to initiate first-line chemotherapy while awaiting EGFR test results, also patients with uncommon or rare EGFR mutations with unknown TKI sensitivity were treated with chemotherapy. For these reasons, the TTNT of the patients treated with first-line chemotherapy was also short in our study. Most of these patients subsequently received TKI therapy. This may also explain why first-line chemotherapy was associated with longer OS than first-generation TKIs in the multivariable analysis. The utilization of first-line chemotherapy for EGFR mutated NSCLCs in Finland has significantly decreased in recent years; it was used in 32% between 2010 and 2016, and in 6% between 2020 and 2023 [14]. The TTNT for second-generation TKIs was short (8.3 months), partly because they were more often used in patients with uncommon EGFR mutations [21], which were associated with poorer TTNT and OS.

In our cohort, 23% of advanced-stage patients had CNS metastases at diagnosis and 38% developed them during follow-up. Patients with CNS metastases were significantly younger, a finding previously reported in Asian studies [22, 23]. OS was worse in patients with CNS metastases than without (5.0 vs. 22.9 months). Among patients with liver metastases, Del19 mutations were associated with longer OS than L858R mutations (22.5 vs. 5.0 months). To our knowledge, this has not been reported previously. Nonetheless, the number of patients in this analysis was small and the follow-up was short. However, given the small number of patients and the relatively short follow-up period, these results should be interpreted with caution and require confirmation in larger cohort studies with longer follow-up.

Bone metastases were present in 38% of advanced-stage patients at diagnosis and were associated with an OS of 13.6 months, similar to findings from a Dutch cohort (bone metastasis frequency: 52%; OS: 15.5 months) [24] and a European real-world study (bone metastasis frequency: 40%) [20]. The OS was similar in patients with or without bone metastases, possibly due to the presence of other metastases, such as CNS or liver disease, in patients without bone metastases. Like CNS metastases, bone metastases were more common in younger patients. Similar findings have been reported earlier [25, 26]. These results suggest that NSCLC may be more aggressive at a younger age, irrespective of EGFR mutation status; however, whether age-related tumor characteristics differ in younger patients requires further research.

The 5-year survival rate in our study for all advanced patients was 15.8%, indicating that only a small proportion of the advanced-stage patients have long-term survival benefit from the treatments used. The 5-year survival of the patients treated with third-generation TKI was not measurable, due to short follow-up (reimbursement start 10/2020), but about half (47.3%) of these patients were alive at 3 years, which is significantly more than with first- (27.7%) or second-generation (30.9%) TKIs.

Our study shows that OS has improved over time with the introduction of third generation TKI for first-line treatment in EGFR-mutated NSCLC. However, resistance also to third-generation TKIs develops inevitably, indicating a need to improve and find novel treatment modes for EGFR-mutated NSCLC. Recent Phase 3 trials have shown better results with first-line combination therapies in EGFR-mutated advanced NSCLC, compared to treatment with osimertinib alone [27, 28].

Our study has limitations. The follow-up period for patients receiving third-generation TKIs was limited, as reimbursement for these treatments in Finland only began in October 2020 and follow-up ended in December 2023. As a result, a large proportion of these patients were still undergoing treatment at the end of the follow-up. Furthermore, all potential confounding variables, including performance status (ECOG) and smoking history were not available for majority of the patients. Several potential sources of bias should be considered when interpreting the OS results, including survivor bias related to the 3-month minimum follow-up requirement, confounding by indication in treatment selection, and calendar-time confounding in comparisons between TKI generations. The number of patients in specific subgroup analyses was low, including those with Exon20ins, Major uncommon, Other uncommon and Multiple EGFR mutation subtypes, as well as Del19 and L858R mutations within the CNS, liver, and bone metastasis groups. The histological information was based on ICD-10 codes rather than individual pathology reports. The study was conducted in two university hospitals Southern Finland, which may limit the generalizability of the results to other regions, countries, healthcare systems and EGFR testing practices.

To our knowledge, this is the first study to comprehensively assess the impact of EGFR mutational subtypes on treatment outcomes in both early- and advanced-stage NSCLC. This study shows that EGFR mutational subtype affects outcomes in advanced-stage but not early-stage NSCLC. The findings highlight the need for better treatments, particularly for patients with L858R mutations, and more broadly, the need to enhance OS in EGFR-mutated advanced NSCLC in general. In addition, careful metastasis assessment in younger patients, especially for CNS metastases is needed.

## Supplementary Material



## Data Availability

Raw data are available upon research permit and data request from national health data access body. The data sharing policy of Johnson & Johnson is available at https://innovativemedicine.jnj.com/our-innovation/clinical-trials/transparency. Analysis of the data for this study were made available by Company NHG and used under license for this study and are not publicly available. Other researchers should contact NHG.
