# Supplementary material for: FIN-EGFR: real-world treatment outcomes by EGFR mutational subtypes in NSCLC in Southern Finland
Source: Acta Oncol. 2026 Sep 23;65:46295. doi: 10.2340/1651-226X.2026.46295 (PMC13615412; doi:10.2340/1651-226X.2026.46295)
Supplement: Supplementary file 1 [file AO-65-46295-s1.pdf]

Supplementary material has been published as submitted. It has not been copyedited, or typeset by Acta Oncologica

Supplementary Table 1: Cox PH regression multivariable results for OS for patients diagnosed at early stage (n=210)

Variable	Category	n (%)	HR	95%, CI	p-value
Sex	Male	62 (29.5)	-	-	-
	Female	148 (70.5)	0.60	0.30-1.19	p=0.144
Age group	<65	53 (25.2)	-	-	-
	65-75	87 (41.4)	1.96	0.76-5.01	p=0.161
	>75	70 (33.3)	1.57	0.50-4.92	p=0.443
EGFR mutation	Del19	86 (41.0)	-	-	-
	L858R	72 (34.3)	1.57	0.72-3.38	p=0.254
	Exon20ins	8 (3.8)	0.70	0.09-5.50	p=0.732
	Major uncommon	15 (7.1)	0.42	0.05-3.32	p=0.412
	Multiple	14 (6.7)	0.40	0.05-3.07	p=0.376
	Other uncommon	15 (7.1)	1.98	0.63-6.28	p=0.245
First therapy	Surgery	138 (65.7)	-	-	-
	Surgery/Radiotherapy +adjuvant/neoadjuvant chemotherapy	30 (14.3)	4.42	1.92-10.21	p=0.001
	Radiotherapy with curative intention	34 (16.2)	3.66	1.31-10.23	p=0.013
	Surgery + 3rd gen TKI adjuvant	8 (3.8)	5.83	0.69-49.06	p=0.104

The proportional hazards assumption was assessed showed no evidence of violation (global p = 0.78). Del19: exon 19 deletions; L858R: exon 21 L858R substitutions; Exon20ins: exon 20 insertion; Multiple: more than one EGFR mutation; Major uncommon mutations: G719X, L861Q, or S768I; Other uncommon: rare EGFR mutations; TKI:tyrosine kinase inhibitor

Supplementary Table 2: Cox PH regression multivariable results for OS for patients diagnosed at early stage and receiving surgery as first therapy (n=138)

Variable	Category	n (%)	HR	95%, CI	p-value
Sex	Male	41 (29.7)	-	-	-
	Female	97 (70.3)	0.53	0.20-1.43	p=0.211
Age group	<65	37 (26.8)	-	-	-
	65-75	65 (47.1)	1.30	0.40-4.24	p=0.661
	>75	36 (26.1)	0.97	0.21-4.45	p=0.971
EGFR mutation	Del19	62 (44.9)	-	-	-
	L858R	48 (34.8)	1.82	0.65-5.09	p=0.253
	Other	28 (20.3)	0.30	0.04-2.43	p=0.257

The proportional hazards assumption was assessed showed no evidence of violation (global p = 0.66). Del19: exon 19 deletions; L858R: exon 21 L858R substitutions; Exon20ins: exon 20 insertion; Multiple: more than one EGFR mutation; Major uncommon mutations: G719X, L861Q, or S768I; Other uncommon: rare EGFR mutations

Supplementary Table 3: Cox PH regression multivariable results for TTNT for patients diagnosed at early stage (n=210)

Variable	Category	n (%)	HR	95%, CI	p-value
Sex	Male	62 (29.5)	-	-	-
	Female	148 (70.5)	0.50	0.32-0.77	p=0.002
Age group	<65	53 (25.2)	-	-	-
	65-75	87 (41.4)	1.31	0.75-2.32	p=0.345
	>75	70 (33.3)	1.36	0.72-2.59	p=0.346
EGFR mutation	Del19	86 (41.0)	-	-	-
	L858R	72 (34.3)	0.97	0.59-1.59	p=0.908
	Exon20ins	8 (3.8)	0.35	0.08-1.49	p=0.155
	Major uncommon	15 (7.1)	0.62	0.24-1.61	p=0.327
	Multiple	14 (6.7)	1.05	0.47-2.36	p=0.905
	Other uncommon	15 (7.1)	1.07	0.47-2.47	p=0.866
First therapy	Surgery	138 (65.7)	-	-	-
	Surgery/Radiotherapy +adjuvant/neoadjuvant chemotherapy	30 (14.3)	2.55	1.46-4.47	p=0.002
	Radiotherapy with curative intention	34 (16.2)	2.45	1.36-4.43	p=0.003
	Surgery + 3rd gen TKI adjuvant	8 (3.8)	0.72	0.10-5.28	p=0.744

The proportional hazards assumption was assessed showed no evidence of violation (global p = 0.88). Del19: exon 19 deletions; L858R: exon 21 L858R substitutions; Exon20ins: exon 20 insertion; Multiple: more than one EGFR mutation; Major uncommon mutations: G719X, L861Q, or S768I; Other uncommon: rare EGFR mutations, TKI: tyrosine kinase inhibitor

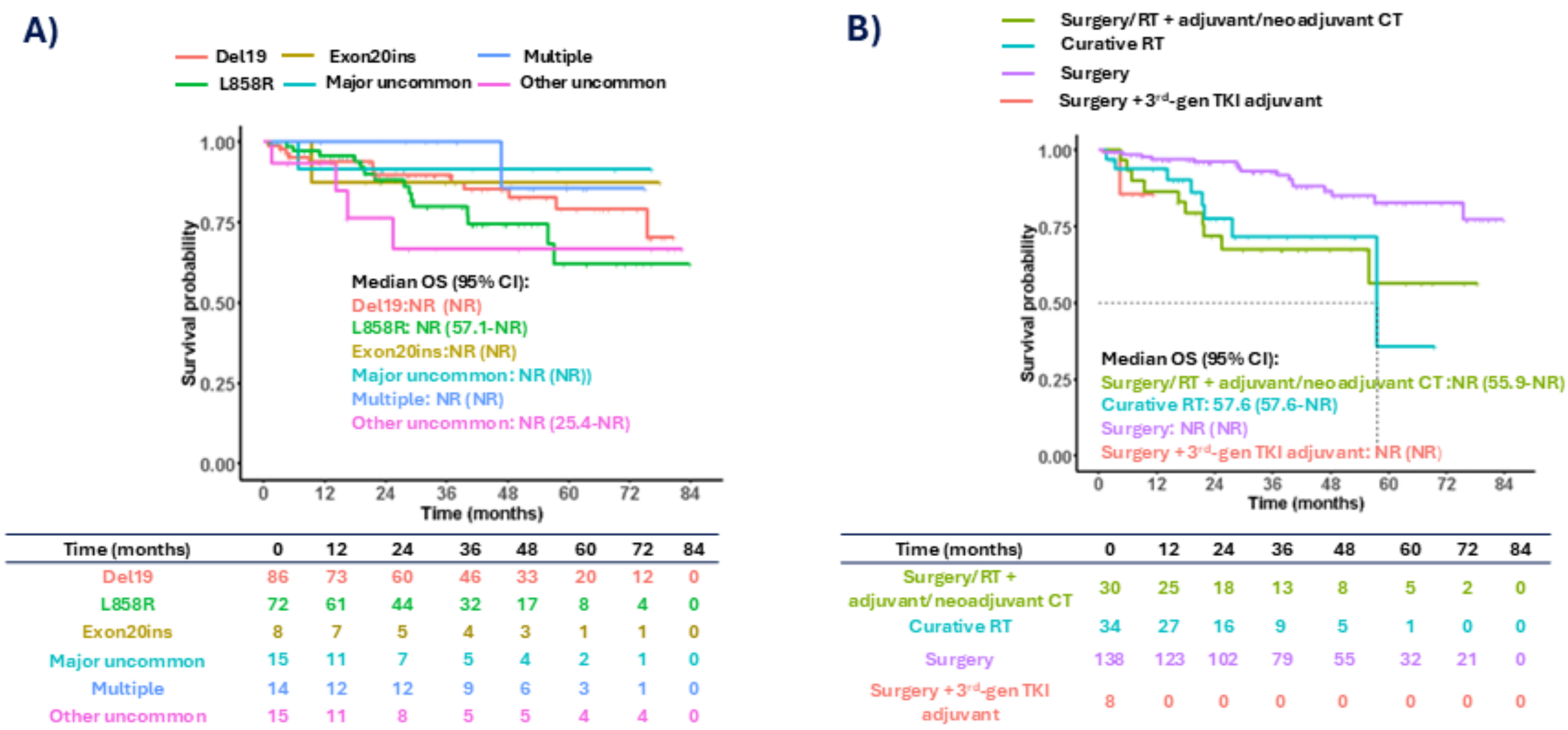

Supplementary Figure 1: OS for patients diagnosed at early-stage EGFR mutated NSCLC by A) EGFR mutational group or B) treatment type. Del19: exon 19 deletions; L858R: exon 21 L858R substitutions; Exon20ins: exon 20 insertion; Multiple: more than one EGFR mutation; Major uncommon mutations: G719X, L861Q, or S768I; Other uncommon: rare EGFR mutations; TKI:tyrosine kinase inhibitor; RT: radiotherapy; NR: not reached

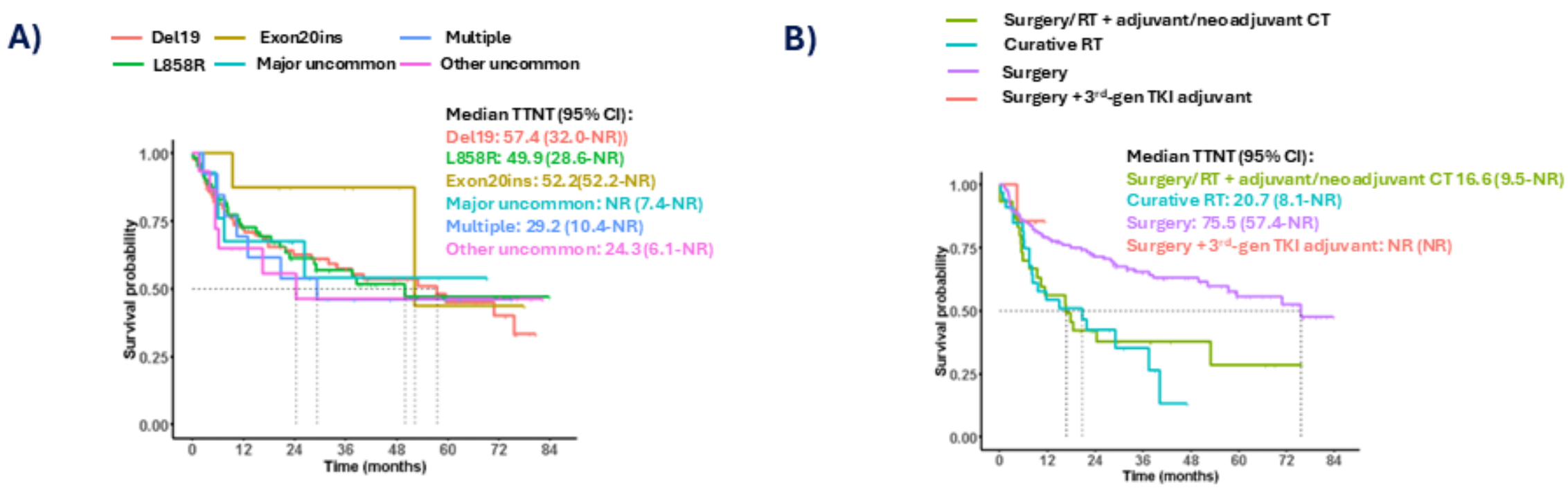

Time (months)	0	12	24	36	48	60	72	84
Del19	86	56	42	30	24	15	8	0
L858R	72	46	29	22	11	5	4	0
Exon20ins	8	7	5	4	3	1	1	0
Major uncommon	15	8	5	3	2	1	0	0
Multiple	14	9	7	5	3	2	1	0
Other uncommon	15	7	6	4	4	3	3	0

Time (months)	0	12	24	36	48	60	72	84
Surgery/ RT + adjuvant/ neoadjuvant CT	30	16	10	8	5	3	1	0
Curative RT	34	16	8	4	0	0	0	0
Surgery	138	101	76	56	42	24	16	0
Surgery +3rd-gen TKI adjuvant	8	0	0	0	0	0	0	0

Supplementary Figure 2: Time to next treatment (TTNT) for patients diagnosed at early-stage EGFR mutated NSCLC by A) EGFR mutational group or B) treatment type. Del19: exon 19 deletions; L858R: exon 21 L858R substitutions; Exon20ins: exon 20 insertion; Multiple: more than one EGFR mutation; Major uncommon mutations: G719X, L861Q, or S768I; Other uncommon: rare EGFR mutations; TKI:tyrosine kinase inhibitor; RT: radiotherapy; NR: not reached
